# Handheld magnetic probe with permanent magnet and Hall sensor for identifying sentinel lymph nodes in breast cancer patients

**DOI:** 10.1038/s41598-018-19480-1

**Published:** 2018-01-19

**Authors:** Masaki Sekino, Akihiro Kuwahata, Tetsu Ookubo, Mikio Shiozawa, Kaichi Ohashi, Miki Kaneko, Itsuro Saito, Yusuke Inoue, Hiroyuki Ohsaki, Hiroyuki Takei, Moriaki Kusakabe

**Affiliations:** 1 Graduate School of Engineering, The University of Tokyo, Tokyo, 113-0032 Japan; 2 Tochigi-Medical-Center-Shimotsuga, Tochigi, 329-4498 Japan; 3 iMed Japan Inc, Chiba, 275-0001 Japan; 4 Institute of Development, Aging and Cancer, Tohoku University, Miyagi, 980-8575 Japan; 5 Graduate School of Frontier Sciences, The University of Tokyo, Kashiwa, 277-8561 Japan; 6 Department of Breast Oncology, Nippon Medical School Hospital, Tokyo, 113-8603 Japan; 7 Research Center for Food Safety, Graduate School of Agricultural and Life Sciences, The University of Tokyo, Tokyo, 113-8657 Japan; 8 Matrix Cell Research Institute Inc, Ibaraki, 300-1232 Japan

## Abstract

The newly developed radioisotope-free technique based on magnetic nanoparticle detection using a magnetic probe is a promising method for sentinel lymph node biopsy. In this study, a novel handheld magnetic probe with a permanent magnet and magnetic sensor is developed to detect the sentinel lymph nodes in breast cancer patients. An outstanding feature of the probe is the precise positioning of the sensor at the magnetic null point of the magnet, leading to highly sensitive measurements unaffected by the strong ambient magnetic fields of the magnet. Numerical and experimental results show that the longitudinal detection length is approximately 10 mm, for 140 μg of iron. Clinical tests were performed, for the first time, using magnetic and blue dye tracers—without radioisotopes—in breast cancer patients to demonstrate the performance of the probe. The nodes were identified through transcutaneous and *ex-vivo* measurements, and the iron accumulation in the nodes was quantitatively revealed. These results show that the handheld magnetic probe is useful in sentinel lymph node biopsy and that magnetic techniques are widely being accepted as future standard methods in medical institutions lacking nuclear medicine facilities.

## Introduction

Sentinel lymph node biopsy (SLNB) is performed to investigate tumor metastases and determine the appropriate sequence of care for breast cancer patients^1–3^. By performing SLNB, breast cancer patients can avoid unnecessary invasive procedures and the resulting side effects such as lymphedema, seroma, and numbness, associated with axillary node dissections. That is, SLNB provides a noninvasive treatment option. In general, breast cancer spreads predominantly via the lymphatic system, and the sentinel lymph node (SLN) is the first lymph node that receives the lymphatic drainage from the primary tumor site. Thus, it is essential to detect the SLNs in the procedure of SLNB. To date, the standard technique for SLN detection is a “combined technique” that uses tracers of radioisotope nanocolloids (generally technetium 99 m: ^99m^Tc) and blue dye, to identify the SLNs with a gamma probe^4,5^ and visual observation^6^, respectively^7–13^. The radioisotope technique, in particular, which has the highest detection rate for SLNs, is based on radiation detection and provides quantitative identification to determine the incision location transcutaneously. However, both methods have significant drawbacks. The visual observation of the blue dye by the surgeon is subject to their own discretion, which makes it a subjective assessment. Moreover, it may cause allegoric/anaphylactic reactions^14^. The radioisotope method may not be accessible for all patients, owing to the lack of radiation facilities in some medical institutions. Furthermore, the use of radioisotope tracers exposes patients as well as technicians to radiation. The development of new techniques to solve these issues in detecting SLNs in a clinical site is thus becoming necessary.

Superparamagnetic iron oxide nanoparticles (SPIONs) are being widely used as contrast agents in magnetic resonance imaging (MRI) in medical institutions without radiation facilities. The particles injected into the human body for localizing the SLNs move into the SLNs via the lymphatic system and can be visualized using MRI. However, the limited availability of MRI facilities remains a key issue; without MRI facilities, only preoperative localization of the SLNs can be performed. This information is insufficient for intraoperative detection, considering that the shape of the breast may change with changes in posture during surgery.

In contrast, a handheld magnetometer can facilitate the intraoperative identification of SLNs by using SPIONs. A handheld magnetic probe with a superconducting quantum interference device (SQUID) provides highly sensitive magnetic detection^15^ and has been used in clinical studies on mice^16–18^. However, it is difficult to introduce the magnetometer into the operation room, as it requires liquid nitrogen/helium as a refrigerant.

A handheld magnetometer that can be used at room temperatures, i.e., without a refrigerant, has been developed as a magnetic technique to detect the SLNs in breast cancer patients. A clinical study using the magnetometer and SPION tracers was performed on breast cancer patients^19^, and subsequent studies revealed that the magnetic technique was not inferior to the radioisotope technique^20–25^, which indicated that the technique could be a prospective standard method without any limitations. A meta-analysis (more that 1000 patients) of SLNB using the magnetic technique has reported that the mean identification rate was 97.1% (range 94.4–98.0)^26^, which is comparable to the radioisotope technique (96.8%). Among the magnetic techniques, there are two common methods used to generate the magnetic field required to magnetize the SPIONs: one uses an electromagnetic coil for generating an alternating magnetic field^20–25^ (as described by the many clinical trials using the Sentimag magnetometer). The other uses a permanent magnet for generating a direct magnetic field^19^ (as demonstrated by M. Shiozawa *et al*.). The benefit of using an electromagnetic coil is that the strength of magnetic field can be adjusted and the nonlinear magnetic characteristics can be utilized^27,28^. However, the disadvantage of this method is that it requires a large power supply to generate strong magnetic fields, which in turn leads to the need for a countermeasure for Joule heating. The relatively large power that is required intrinsically implies that the probe cannot be constructed without a power cable and power unit, which is a limitation (e.g., lack of the transportability and controllability) for installation into clinical sites to some extent. By contrast, the use of a magnet that requires the generation of moderately strong fields, in the order of 100 mT without the electricity, promoted the creation of the compact handheld magnetic probe system without the power cable, which facilitates easier intraoperative detection because of its small size.

**Figure 1:**
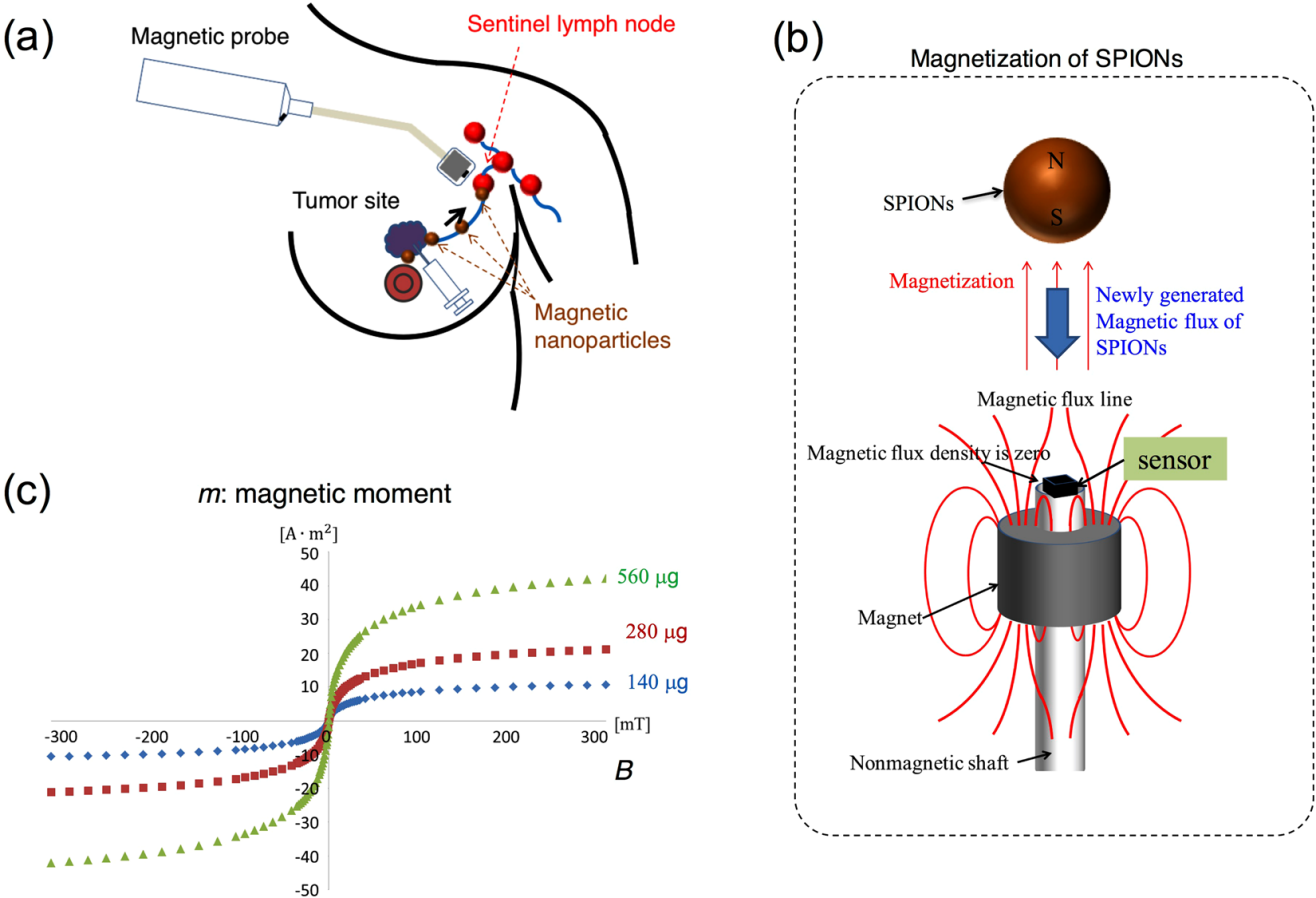
(**a**) Principle of the technique based on magnetic detection of SLNs using a handheld magnetic probe and magnetic nanoparticles for breast cancer patients. Injected magnetic nanoparticles accumulate in the SLNs via the axillary lymphatic system and are detected by a magnetometer. (**b**) Schematic of the magnetic field lines produced by the ring-shaped permanent magnet. There are two magnetic null points on the surface of the magnet along the axis. The magnetic sensor is located at a magnetic null point. (**c**) Superparamagnetic characteristics of Resovist (Ferucarbotran); the magnetic moment measured by the SQUID apparatus as a function of the applied magnetic field from −300 to 300 mT.

Obviously, while larger magnets can generate stronger magnetic fields, large-sized probes face issues related to transportability and controllability. To establish the magnetic technique using a permanent magnet, the optimum magnet size in the probe must be verified. In this study, numerical and experimental evaluations are performed to optimize the shape and size of the magnet and to reveal the sensitivity of the probe. We developed a novel handheld magnetic probe with a permanent magnet and a magnetic sensor for identifying the SLNs. This probe incorporated a unique outstanding feature for high-sensitivity measurements and displayed the measured magnetic fields quantitatively in units of μT. We performed the first ever clinical test with SPIONs and blue dye (without radioisotopes) tracers to demonstrate the performance of the prototype magnetic probe in SLNB for breast cancer patients. Figure 1(a) shows the principles behind the identification of SLNs using a handheld magnetic probe with a permanent magnet and magnetic sensor, which is a promising nonradioisotope method based on magnetic detection for breast cancer patients. The SPIONs are injected into the vicinity of the cancer lesion (e.g., subareolar, subcutaneous, or intradermal site), and they move into the SLNs through the lymph vessels. The SPIO tracers accumulated in the SLNs are magnetized by the magnetic fields of the permanent magnet enclosed in the head of the magnetic probe (Fig. 1(b)), and subsequently, the SLNs containing the magnetic nanoparticles are identified by measuring the magnetic fields newly generated from the magnetized nanoparticles^19,20,29–32^.

## Results and Discussion

### Magnetic technique for the SLNB using a handheld magnetic probe with a permanent magnet and magnetic sensor

Figure 1(b) illustrates the magnetic field lines of the ring-shaped permanent magnet (neodymium magnet, NF40) enclosed in the head of the magnetic probe. There are two magnetic null points where the magnetic flux density is zero: at the top and bottom of the magnet. A unique outstanding feature of this developed magnetic probe is that the magnetic sensor is located at the magnetic null point at the top of the magnet; thus, tiny variations in the magnetic fields can be detected without the offset attributes from the nonzero magnetic fields. Thus, the amplifier gain can be enhanced optimally for tiny magnetic signals

Figure 1(c) shows the magnetic characteristics of Resovist^33^ (magnetic fluid containing the SPIONs) measured using the SQUID MPMS-5S, Quantum Design, Inc., US, for various iron amount 140, 280, and 560 μg, corresponding to 5, 10, and 20 μL of Resovist, respectively. Increasing the applied magnetic field strongly increases the magnetic moment of the SPIONs in the range of 0–50 mT, and the measured magnetic moments are clearly proporonal to their amounts. The SPIONs can be fully magnetized by applying a magnetic field of approximately 50 mT. These results show that the maximum magnetic susceptibility χ_max_ is approximately 0.16. Here, the relation between *B* and *H* is $B={\mu }_{0}{\mu }_{r}H={\mu }_{0}(1+\,\chi )H$, where *B* is the strength of the magnetic field, *H* is the magnetic field, ${\mu }_{r}$. is the relative permeability, and *μ*_0_ is the permeability of free space. Thus, the relative permeability of the SPION is approximately 1.16. In the presence of a magnetic field of 50 mT, the magnetization of the magnetically saturated SPIO is approximately $M=\chi H=1.5\times {10}^{3}{\rm{A}}/{\rm{m}}$.

### Numerical evaluation of the characteristics of a handheld magnetic probe

In the SLNB using the magnetic technique, a permanent magnet and magnetic particles generate the magnetic fields. To evaluate the spatial distributions of the magnetic fields and the sensitivity characteristics of the magnetic probe, numerical calculations are carried out using the finite element method (FEM) (the detailed method is shown in supplementary Fig. S1).

**Figure 2:**
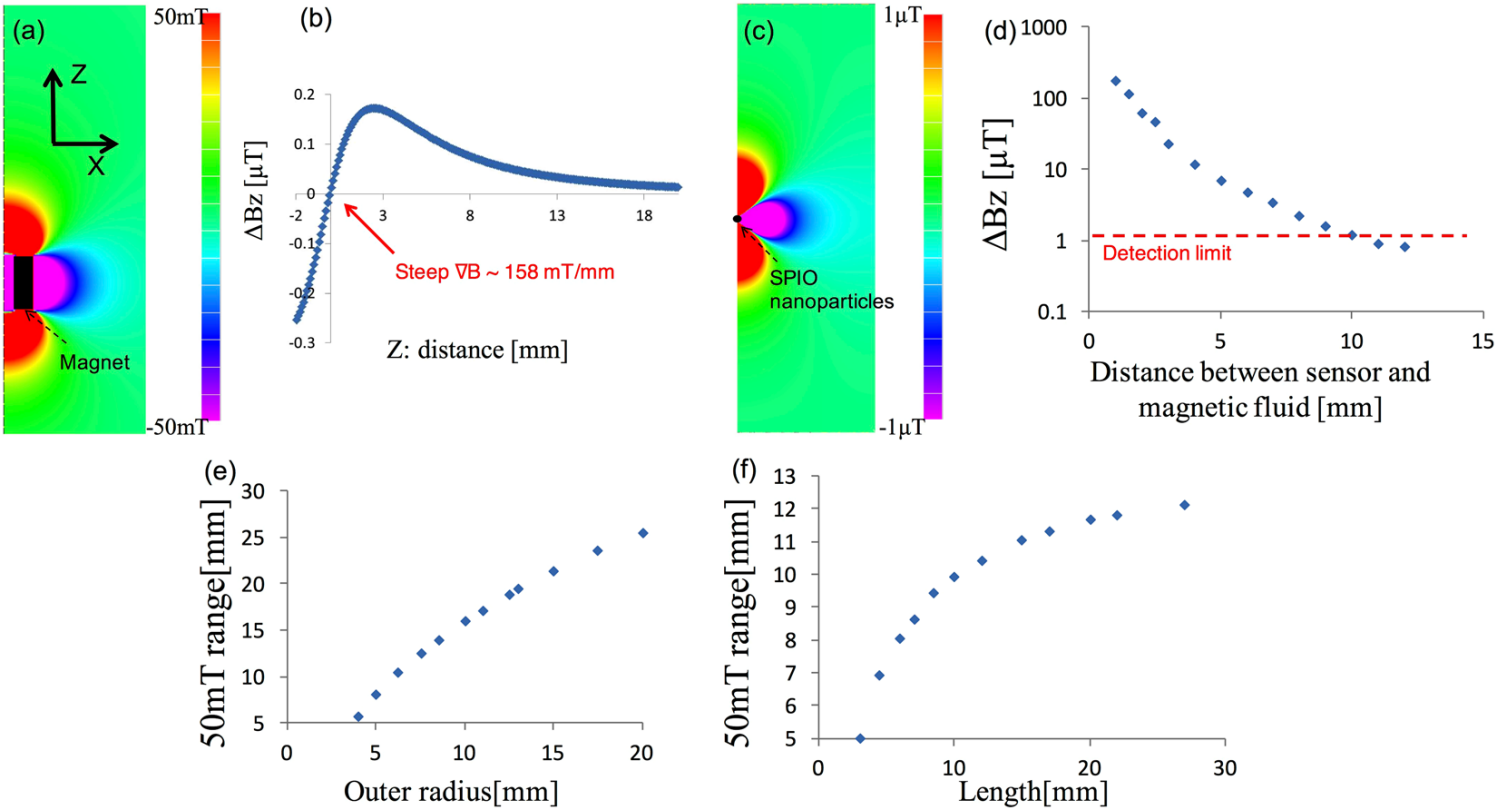
Numerical results: (**a**) Two-dimensional distribution of the longitudinal magnetic field *B*_*Z*_ of the permanent magnet. (**b**) *B*_*Z*_ of the magnet on the *Z*-axis (*X* = 0 mm). *∇B* (*dB*_*Z*_*/dz)* is approximately 158 mT/mm around the magnetic null point (*Z* = 0.3 mm). (**c**) Two-dimensional distribution of *B*_*Z*_ of 140 μg of iron (5 μL of Resovist) of fully magnetized SPIO located at (*X, Z*) = (0, 10.4) mm. (**d**) Magnetic field strength Δ*B* at the sensor position as a function of the distance from the magnetic sensor to the SPIONs location on the *Z*-axis. Red dotted line represents the sensor detection limit of 1 μT. Relationships between (**e**) the outer radius and (**f**) the length of the magnet, and the distance *Z*_50_ of the point where the *B*_*Z*_ of the magnet is 50 mT from the magnet surface.

The two-dimensional distribution of the calculated longitudinal magnetic field (*B*_*Z*_) generated by the permanent magnet (outer radius and length are 6.25 and 12 mm, respectively) is shown in Fig. 2(a) with a color contour range of ±50 mT. At *Z* = 10.4 mm on the probe axis (*Z*-axis, *X* = 0 mm), the strength of the magnetic field *B*_*Z*_ is approximately 50 mT and the position of the magnetic null point is *Z* = 0.3 mm, as shown in Fig. 2(b). Figure 2(c) shows the distributions of the calculated magnetic fields *B*_*Z*_, with a contour range of ±1 μT, generated by 140 μg of iron (5 μL of Resovist) of magnetically saturated SPIONs, located at *Z* = 10.4 mm. Figure 2(d) shows that the strength of the magnetic field, Δ*B*, at the sensor position (magnetic null point) depends on the distance between the sensor and the SPIO position. The magnetic signal from the SPIONs decreases with an increase in distance: the magnetic fields fall strongly to 14% between 5 and 10 mm. For the actual use, the current sensitivity limit of the Hall sensor (NHE-520) is 1 μT as shown in Fig. 2(d), owing to the noise attributed to thermal fluctuations. Thus, the present sensor can detect magnetic signals of 140 μg of SPIONs located at a distance of 10.4 mm, which means that “the longitudinal detectable distance” is approximately 10 mm. We should also note that, there are sensors with higher sensitivities, which work at room temperature, such as the giant magneto-resistance (GMR) and magneto-impedance (MI) sensors. However, these sensors do not work successfully in the vicinity of a magnet because their magnetic characteristics may change due to the nonuniformity of the strong magnetic field^28,29^.

To determine the optimum size of the magnet, we evaluated the distance range for a field of 50 mT (*Z*_50_) generated by varying the size of the magnet for the full magnetization of SPIONs. Figure 2(e) and (f) show the relationship between the outer radius and the length of the magnet and the distance *Z*_50_ of the point where the longitudinal magnetic field, *B*_*Z*_, is 50 mT from the magnet surface, respectively. The distance *Z*_50_ increases linearly as the outer radius of the magnet increases. On the other hand, the increment of *Z*_50_ as a function of the magnet length saturates roughly at 12 mm length of the magnet. The result indicates that it is more effective to increase the outer radius rather than the length of the magnet for increasing the distance *Z*_50_. However, Resovist is sufficiently magnetized by a magnetic field of 50 mT. For example, even if the Resovist that is magnetically saturated under the strong magnetic field is located at a distance of 20 mm (in the case of the outer radius is 13 mm as shown in Fig. 2(e)), the magnetic signal from the Resovist is less than 1 μT, indicating that the signal cannot be detected by the Hall sensor. Moreover, a large magnet is associated with poor transportability and controllability. Thus, the optimum outer radius and length of the magnet are considered to be 6.25 mm and 12 mm, respectively, taking into account that producing a 50 mT at the distance of 10 mm away is sufficient to detect the SPIONs.

### Development of a prototype handheld magnetic probe and experimental evaluation of sensitivity characteristics

We developed a handheld prototype of a magnetic probe with a permanent magnet and Hall sensor and evaluated its sensitivity experimentally. As described above, it is essential that the sensor is located correctly at the magnetic null point, for obtaining high-sensitivity measurements without the offsets caused by the background magnetic fields, which is a unique outstanding feature of our magnetic probe. However, the spatial variations of the magnetic fields are too steep around the magnetic null point, 158 mT/mm [see Fig. 2(b)]. We utilize a newly developed apparatus (see supplementary Fig. S2) to arrange the sensors with spatial resolutions of less than a few micrometers; Consequently, the Hall element inside the sensor can be fixed at the magnetic null point correctly, with a field resolution of 1 μT, which is almost equivalent to 0.01 μm.

**Figure 3:**
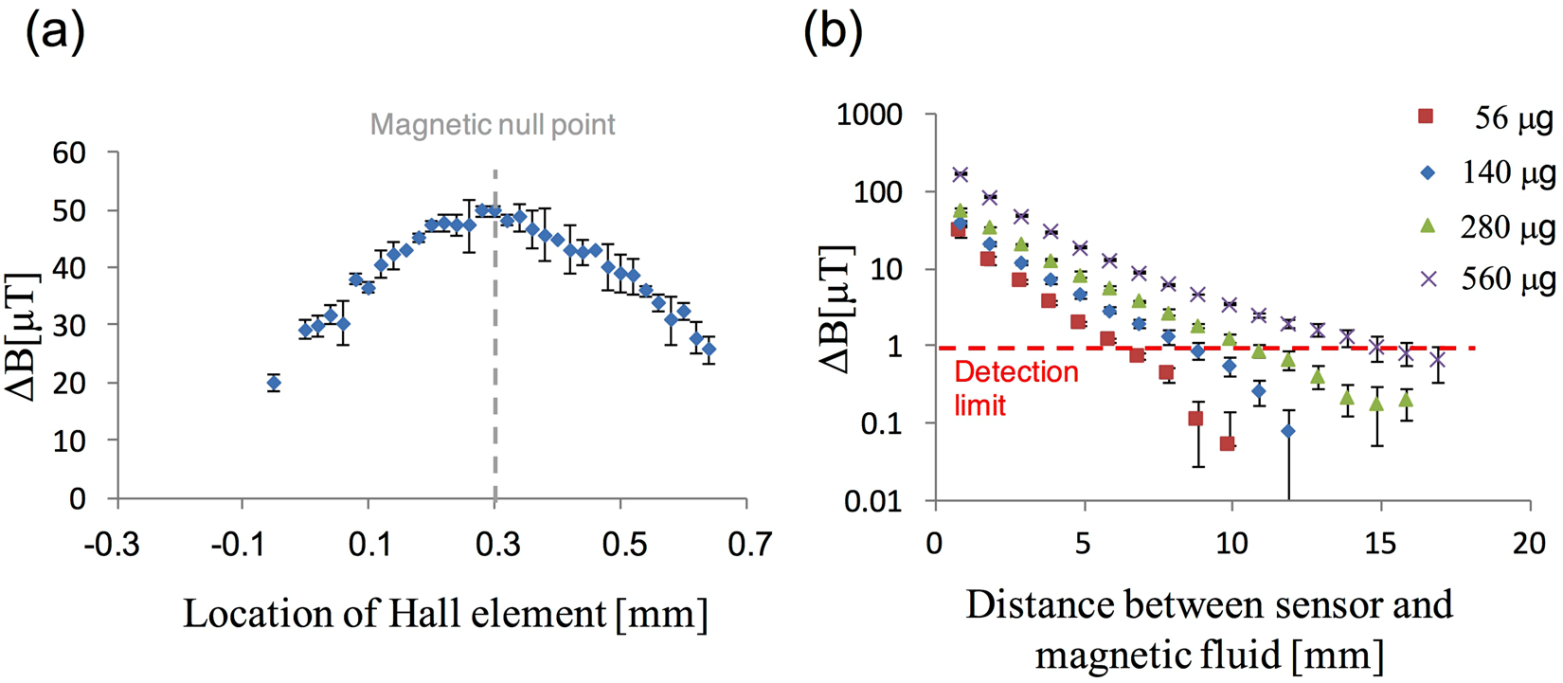
(**a**) Signals detected with 140 μg of iron (5 μL of Resovist), as functions of the Hall element position: the gray dotted line shows that the magnetic null point is at approximately 0.3 mm. (**b**) Measured signals for four different volumes, 56, 140, 280, and 560 μg of iron. The red dotted line shows the detection limit of the sensor.

Figure 3(a) shows the detected signal dependence on the positional displacement of the Hall element inside the sensor. At approximately 0.3 mm—the magnetic null point (Fig. 2(b))— the detected signal reaches its highest value, reading 50 μT, for 140 μg of iron. When the Hall sensor position deviates from the magnetic null point, the detected signal decreases gradually because of the poor linearity of the InSb Hall element in the presence of strong magnetic fields (approximately 20 mT) as shown in Fig. 3(a). The spatial gradient of the magnetic field around the magnetic null point is approximately 158 mT/mm, as shown in Fig. 2(b). Thus, the sensor must be fixed within the error of ±0.1 mm, which is equivalent to ±15.8 mT, to maintain its high sensitivity. To investigate the manufacturing error, that is the reproducibility, we made five probes. As a result, the error was 2 ± 3 μm (a few mT) and was sufficiently small to obtain highly sensitive measurements. The previous magnetometer that consisted a permanent magnet and permalloy has a similar feature that in the sensor is located at the magnetic null point by utilizing the arrangement of the inner and outer permalloy, and the clinical study using the probe was successful for the detection of the nodes^19^. However, there was no reproducibility, to some extent, regarding the correct arrangement of the sensor location relatively to the inner, outer permalloy, and magnet. Therefore, we developed the simple magnet configuration without permalloy in this study to establish the correct arrangement of the null point at the sensor location.

Figure 3(b) shows the detected signals from different amounts of SPIONs, 56, 140, 280, and 560 μg, as functions of the distance between the surface of the probe head and the SPIONs. The measured magnetic flux density increased with increasing the amount of SPIONs, and the detectable length was 7, 9, 11, and 15 mm for 56, 140, 280, and 560 μg, respectively, which also agrees well with the numerical results as shown in Fig. 2(d), by taking into account the distance between the sensor and the surface of the probe head [see supplementary Fig. S3a].

### Clinical trials with SPIONs and blue dye tracers

**Figure 4:**
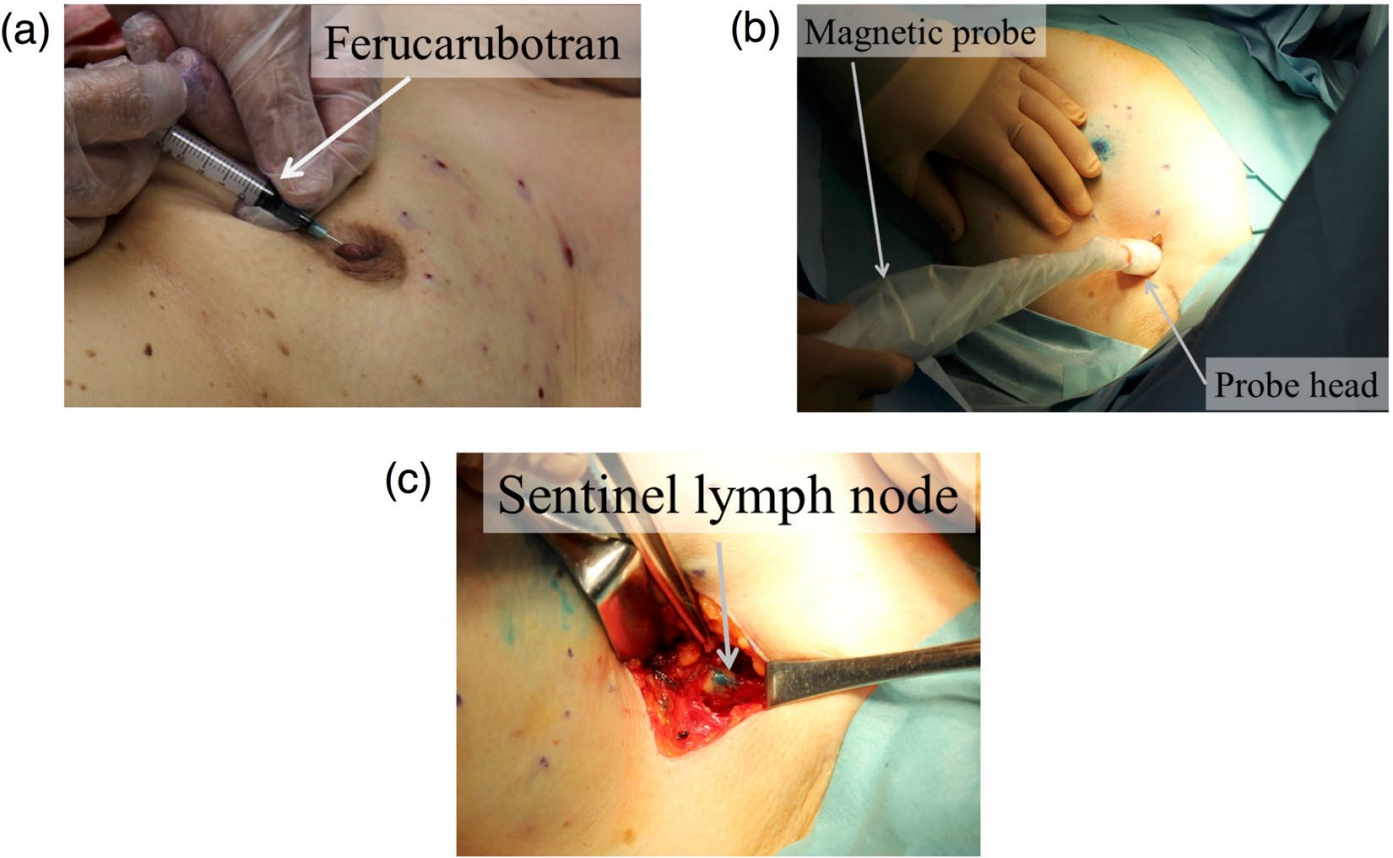
Procedure of the clinical test for the magnetic technique involving SPIO and blue dye tracers, using the handheld magnetic probe with a permanent magnet and Hall sensor for SLNB; (**a**) injection of SPIO tracers around the subareolar region, (**b**) transcutaneous detection of the SLNs before incision, and (**c**) SLNs detection and excision in the axilla after incision. In the injection phase (**a**), the blue dye tracer is injected at the same location after the injection of the SPIO tracer.

We performed a small clinical trial on six breast cancer patients in the Sin-Oyama City Hospital in Japan, in accordance with the protocols approved by the clinical community, to verify the feasibility of the developed handheld magnetic probe (see supplementary Fig. S3c). SPIONs (Resovist, 1.6 mL) and blue dye tracers (Patent Blue V, 3 mL) were injected into the subareolar region as shown in Fig. 4(a), as per a new combined technique without radioisotopes. After injection, surgeons massaged the vicinity of the injection site for 10 min to promote the lymphatic flow and tracer accumulation. The surgeons subsequently performed transcutaneous detection of the SLNs using the magnetic probe to determine the resection location, as shown in Fig. 4(b); the strength of the magnetic signal was approximately 1–2 μT in the transcutaneous detection. Eventually, the surgeons succeeded in finding the SLNs by magnetic detection using the probe and visual observation of the blue and/or brown colored nodes and excised the nodes as shown in Fig. 4(c). After extracting the SLNs, they confirmed that there were no SLNs in the axilla through scanning with the magnetic probe; the measured magnetic signal was less than 1 μT (the detection limit). The SLNs were successfully detected using the magnetic technique in four patients. The strength of the magnetic signal from the nodes was approximately 11 ± 5 μT *in situ* and the *ex vivo* measurements. Out of these four cases, we succeeded in the transcutaneous detection of the lymph nodes in three cases. In the other case, the iron amount measured by SQUID was approximately 30 μg (1.1 μL of Resovist), which was too small to be detected from the skin surface using the prototype magnetic probe. Because the iron accumulation and node location strongly depend on the patient’s characteristics such as age and BMI, it may not be possible to detect the nodes using the prototype probe in some cases. Currently, it is preferred that the probe is utilized for identifying the SLNs using in/*ex vivo* measurements rather than transcutaneous measurement. Further studies are required to improve the sensitivity of the probe and the strong magnetization of the nanoparticles.

**Figure 5:**
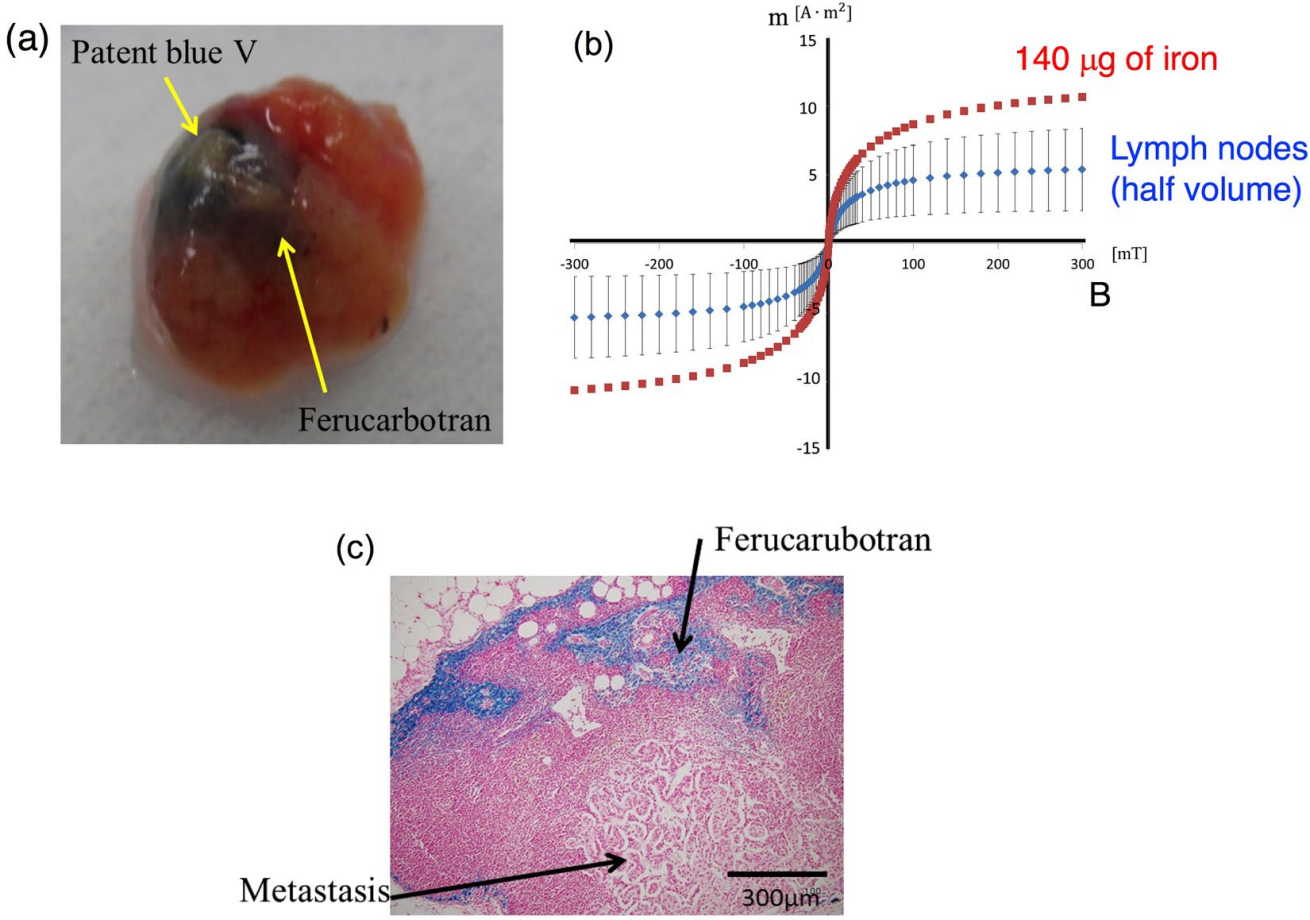
(**a**) Blue color of Patent blue and brown color of Resovist (Ferucarbotran) in the excised node. (**b**) Magnetic moments of a half volume of the excised node and 140 μg of SPIONs measured by the SQUID apparatus. (**c**) Histopathology of the excised node. The distribution of iron staining (Perl’s Prussian blue) is generally in the cortex of the node. Metastasis was observed in one excised node.

It is clearly found that the blue and brown colors in an example of the excised node are caused by the blue dye and Resovist as shown in Fig. 5(a). To evaluate correctly the accumulation of SPIONs in the six excised lymph nodes detected by the magnetic probe from four patients, the magnetic moments of the nodes were measured using the SQUID apparatus as shown in Fig. 5(b). Note that the volume of the measured SLN is half of the volume of the excised nodes, and consequently, the amount of iron in the excised node is double the value calculated from Fig. 5(b). By comparing with the SPIO sample of 140 μg of iron, it is revealed that the amount of iron contained in the excised SLNs is approximately 140 ± 80 μg of iron (5 ± 3 μL of Resovist); 210 240, 77, 30, 200, and 91 μg for each SLN. We also revealed, for the first time, that the iron content contained in the SLNs of breast cancer patients is approximately 0.3% of the injection volume of 1.6 mL (44.6 mg of iron). This is an important knowledge to optimize the injection amount of magnetic nanoparticles for future operations. The large amount of injection would cause the huge susceptibility artifact around the injection site on magnetic resonance imaging (MRI) after surgical operations, which is a drawback in SLNB using magnetic nanoparticles^34^.

The magnetization of the excised nodes as shown in Fig. 5(b) are not completely consistent with the reference data (140 μg of iron), and the difference is contributed by the diamagnetic component of the biomedical tissues. For the other reason, the magnetic characteristics of the nanoparticles might change in the lymph node because the aggregation or disaggregation of the particle may occur in the lymph node. Further research is need to obtain the clear evidence, e.g., the particle concentration.

Figure 5(c) shows the histopathology of the extracted node. The blue area shows the iron deposition, and it is localized in the cortex of the lymph node. The metastasis is also confirmed in one node. It is reported that the SPIO hardly accumulates in the node containing the metastasis^35^. However, according to the clinical trials performed by J.-L. Houepeau *et al*.^25^, the detection rate of the normal nodes (~97%) was almost consistent with that of the carcinogenic nodes (~98%), which suggests that the sentinel nodes contains the sufficient amount of iron to detect the magnetic probe even if there is metastasis in the nodes. That node was detected in this study as well as previous studies.

To further investigate the distribution of the SPIONs in the SLNs, we measured the magnetic signal from a large phantom that simulates the size of the SLNs: the typical long and short-axis lengths of the SLNs are 12.4 and 7.4 mm, respectively^36^, and their volume is approximately 350 μL (Resovist 5 μL and saline 345 μL). In the *ex vivo* measurements, the magnetic signals detected from the small (Resovist 5 μL only) and the large phantom are 40 and 5 μT, respectively. Meanwhile, when the magnetic probe is in contact with the excised SLNs, the measured signal is 11 ± 5 μT, which is larger than the signal detected from the diluted 350 μL of large phantom and is smaller than the signal from the 5 μL of small phantom. Therefore, the SPIONs may be located on the surface of the SLNs, instead of being distributed uniformly within the SLNs and/or localized in the centers of the SLNs, as shown in Fig. 4(c).

## Conclusions

We developed a novel handheld magnetic probe with a permanent magnet and a Hall magnetic sensor to detect the SLNs containing SPIONs in breast cancer patients. An outstanding feature of the probe was the correct positioning of the sensor with respect to the magnetic null point of the permanent magnet, which resulted in highly sensitive measurements without the influence of ambient strong magnetic fields from the neodymium magnet. Numerical and experimental results showed the magnetic probe could detect the magnetic signal of the SPIONs of 140 μg of iron in 5 μL of Resovist when separated by a distance of 10 mm, i.e., the longitudinal detection length was 10 mm. We conducted the first ever clinical study on the combined use of magnetic and blue dye tracers (without radioisotope) for breast cancer patients in the Sin-Oyama City Hospital, to verify the performance of magnetic detection using the magnetic probe. We successfully detected the SLNs containing the magnetic nanoparticles through transcutaneous and *ex vivo* measurements. The newly developed magnetic probe was found to be beneficial for the identification of the SLNs. The clinical test also revealed that the uptake of the magnetic nanoparticles into the nodes was approximately 140 ± 80 μg, which was equivalent to 0.3% of the injection amount of 44.6 mg (1.6 mL of Resovist). Moreover, the SPIONs were mostly localized in the cortex of the nodes.

Although the prototype magnetic probe has a cable for the connection of the measurement and driving circuits, in the near future, we plan to further develop our probe and make it more compact without the cable and small unit by merging the probe body and the driving circuit, because the probe does not require a large power supply for the generation of magnetic fields. This will facilitate straightforward installation in all medical institutions with/without nuclear facilities^30,31^. We believe that this is the main strength of our handheld magnetic probe compared to a magnetometer that uses an electromagnet coil (e.g., Sentimag magnetometer^20–25^). The magnetic technique provides a minimally invasive method for the SLN localization in cancer patients, thereby, improving the quality of life of the patients.

## Material and Methods

### Resovist (magnetic fluid containing SPIONs)

The SPIONs injected into the patient’s body must be established safe for humans. The SPIO tracer we employ is a ferucarbotran, Resovist^32^ (44.6 mg of iron in 1.6 mL vial: iron concentration is approximately 28 mg/mL), Bayer Schering Pharma, DE, which was originally developed as a contrast agent for use in MRI. Resovist consists of a carboxydextran shell to stabilize the magnetic nanoparticle aggregations, and its size is approximately 60 nm^37^. Therefore, the magnetic tracers are suited for accumulation in the lymph nodes via the axillary lymphatic system: the SPIONs accumulates into the sentinel node via lymph vessels because macrophages in the nodes capture the nanoparticles, although it does not have the functionalization to attach actively to carcinogenic cells.

### Numerical simulations

Postprocessor (FEMAP; Numerical Simulation Tech Co., Ltd., JP) and solver (PHOTO-Series: Photon Co., Ltd., JP) were used in the FEM to create the model and perform the numerical analysis (see supplementary Fig. S1). The simulation parameters are as follows. The inner radius and outer radius of the ring-shaped permanent magnet are 2.5 mm and 6.25 mm (varied outer radii are used in Fig. 2 (e)), respectively. The inner radius of the magnet is 2.5 mm and the length of the magnet is 12 mm (different lengths are used in Fig. 2 (f)). The nanoparticle, equivalent to a volume of 5 μL, is a sphere of radius 1.05 mm. The relative permeability of the Neodymium magnet, SPIONs (Resovist), and air are 1.05 (standard value), 1.16 (from Fig. 2(c)), and 1, respectively, remain constant. The coercive forces of the magnet and SPIONs are 1000 (standard value) and 1.5 kA/m (from Fig. 2(c)), respectively. The total element number is approximately 500,000 and the maximum spatial resolution (the minimum element size) is 0.1 mm. Note that the element size increases with the increase in the distance from the magnet and SPIONs: the sizes close to the magnet and SPIONs are approximately 0.1 mm, and the element sizes around the simulation boundaries are approximately 1 mm. To obtain accurate numerical results, the simulation area should be sufficiently large, (*X*, *Z*) = (0–300, −300–300 mm) compared to the sizes of the magnet and SPIONs. The existence of parallel magnetic fields alone at the boundary can be allowed in the initial state of the calculations, which is the boundary condition for the numerical simulations.

### Handheld magnetic probe

A neodymium magnet (NF40, with typical coercive force of approximately 950–1000 kA/m) manufactured by Sagami Chemical Metal Co., Ltd. was used for the magnetization of the SPIONs. The nonmagnetic probe shaft made of brass was inserted into the center hollow of the ring-shaped magnet. The Hall sensor (NHE520, high-output–type Hall element using evaporated InSb film) was fixed using glue on the edge of the shaft and the Hall element in the sensor was located at the magnetic null point. Note that the InSb Hall element is usually used for high-sensitivity measurements in low magnetic fields (typically less than 20 mT). The diameter of the probe head was 18 mm, and the probe housing was made of acrylonitrile-butadiene-styrene (ABS) resin. Conventional electric circuits were used for the measurement of the magnetic signals. The detected signals were displayed on the panel in real time during the intraoperative identification and *in vitro* experiments (see supplementary Fig. S3).

### Measurements using magnetic phantom for evaluating the developed probe

A carboxydextran-coated SPION, ferucarbotran^32^, was employed for use in small magnetic phantoms with iron amounts 140, 280, and 560 μg (volumes of 5–20 μL) for *in vitro* characterization. The SPIONs were fixed with glue in small plastic containers and the magnetic phantoms of various volumes were constructed. The probe head was fixed and the SPIO phantom could be displaced using the linear XY stage. The driving voltage of the Hall sensor was 1 V (current was approximately 20 mA) and the amplifier (CA461F2, NF Corporation, JP) gain was 40 dB during the measurements. To simulate the SLNs of breast cancer patients, a larger size phantom was fabricated. The spherical phantom of 350 μL, which simulated the typical sizes of SLNs with long and short-axis lengths of 12.4 and 7.4 mm, were constructed using 5 μL of Resovist and 345 μL of saline.

### Clinical tests and evaluations of SLN**s**

To demonstrate the performance of the developed magnetic probe system, clinical tests were conducted on six breast cancer patients, whose average age was 69.3 ± 6.7, in accordance with the protocols for the care of patients and scientific purposes. The protocols were approved by the ethical community of the Shin-Oyama City Hospital (Date of registration: 15/10/2009) and the clinical trial was retrospectively registered within UMIN (University Hospital Medical Information Network)-CTR in Japan (Date of registration: 01/12/2017, Registration number: UMIN 000030127). UMIN-CTR is the registry that currently meet the criteria of the WHO registry network. All study participants provided informed consent. Resovist (SPION; 1.6 mL of magnetic fluid containing 44.6 mg of iron) and Patent blue V (blue dye; 1%, 3 mL) were injected (after induction of anesthesia) into the subareolar region in all breast cancer patients participating in the tests. If surgeons could not detect the node using the magnetic probe transcutaneously, the node location was estimated with the traditional method; the resection location was determined with an ultrasound measurement and/or surgeon’s estimation (the locations are typically 1-2 cm below the hairline in the axilla). After the estimation, the surgeon resected the skin and detected the node inside the axilla using the magnetic probe. Excised nodes were measured using the SQUID apparatus, for the quantitative evaluation of the uptake of SPIONs in the SLNs. Histopathology of the lymph nodes was performed after the surgical operation. The extracted formalin-fixed lymph nodes were processed by thin slicing and paraffin wax embedding. The iron deposition and tumor metastasis in the nodes were then assessed using Perl’s Prussian blue (Sigma-Aldrich, Poole, UK) staining and Hematoxylin and Eosin (H&E) staining, respectively.

## Electronic supplementary material

Supplementary information
