# Supplementary material for: Handheld magnetic probe with permanent magnet and Hall sensor for identifying sentinel lymph nodes in breast cancer patients
Source: Sci Rep. 2018 Jan 19;8:1195. doi: 10.1038/s41598-018-19480-1 (PMC5775278; doi:10.1038/s41598-018-19480-1)
Supplement: Supplementary file 1 — Supplementary information [file 41598_2018_19480_MOESM1_ESM.pdf]

Supplementary information

Handheld magnetic probe with permanent magnet and Hall sensor for identifying sentinel lymph nodes in breast cancer patients

Masaki Sekino[1*], Akihiro Kuwahata[1*], Tetsu Ookubo[1], Mikio Shiozawa[2], Kaichi Ohashi[1], Miki Kaneko[1], Itsuro Saito[3], Yusuke Inoue[1,4], Hiroyuki Ohsaki[5], Hiroyuki Takei[6], and Moriaki Kusakabe[7,8]

[1]Graduate School of Engineering, The University of Tokyo, Tokyo 113-0032, Japan
[2]Tochigi-Medical-Center-Shimotsuga, Tochigi 329-4498, Japan
[3]iMed Japan Inc., Chiba, 275-0001, Japan
[4]Institute of Development, Aging and Cancer, Tohoku University, Miyagi, 980-8575, Japan
[5]Graduate School of Frontier Sciences, The University of Tokyo, Kashiwa 277-8561, Japan
[6]Department of Breast Oncology, Nippon Medical School Hospital, Tokyo 113-8603, Japan
[7]Research Center for Food Safety, Graduate School of Agricultural and Life Sciences, The University of Tokyo, Tokyo 113-8657, Japan
[8]Matrix Cell Research Institute Inc., Ibaraki 300-1232, Japan

*These authors contributed equally to this work.
Correspondence and requests for materials should be addressed to M. S. (sekino@bee.t.u-tokyo.ac.jp) or A. K (kuwahata@bee.t.u-tokyo.ac.jp).

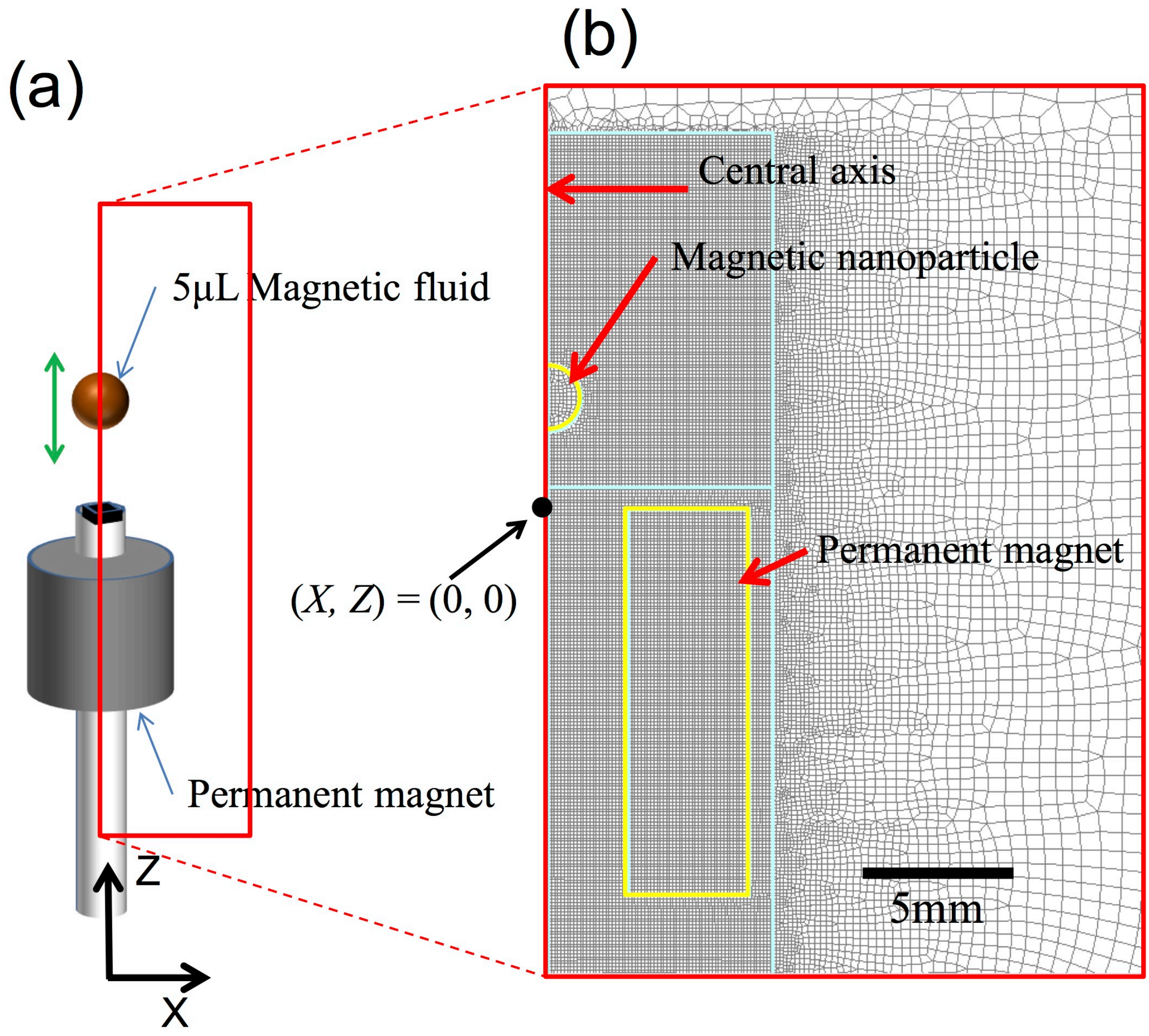

Supplementary Figure S1. Numerical setup using FEM, to evaluate the spatial distributions of the magnetic fields.

Longitudinal and lateral directions with respect to the probe axis are the Z- and X-directions, respectively. Numerical simulations were performed in the two-dimensional (on the X-Z plane) axial symmetric system with respect to the Z-axis. (a) Schematics of the magnetic probe and SPIONs. (b) Numerical simulation model; black solid lines represent grid lines formed the numerical elements in the FEM. Denser lines indicate smaller numerical elements in space to obtain the accurate numerical results in the vicinity of the magnet and nanoparticles. Areas enclosed by yellow solid lines indicate the locations of the SPIONs and the magnet. The magnet surface is located at $Z = 0$ mm.

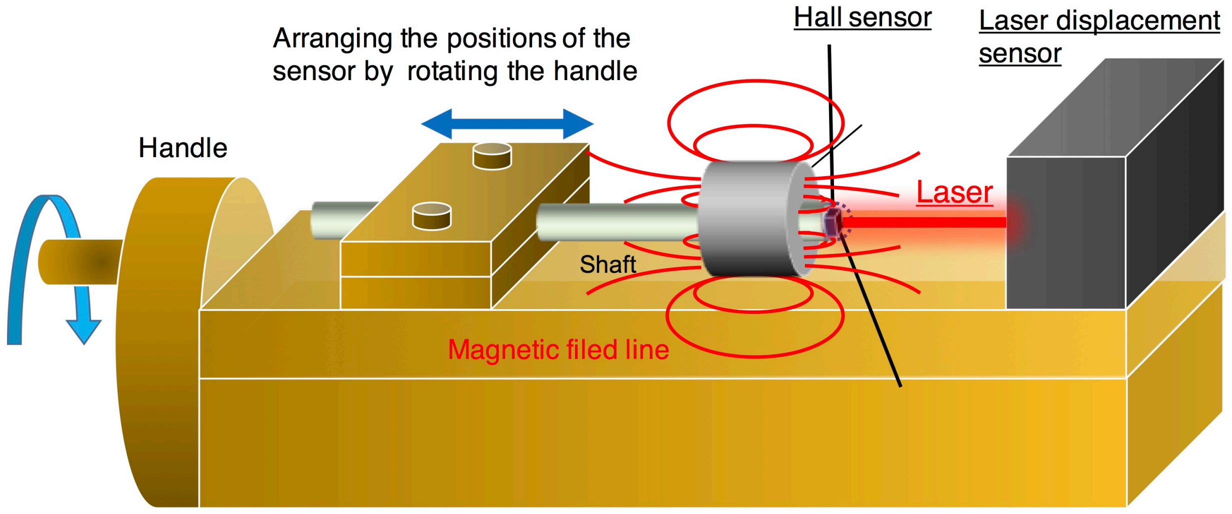

Supplementary Figure S2. Fabrication equipment for the head of the magnetic probe.

Apparatus for adjusting the position between the permanent magnet and Hall sensor with a spatial resolution in the order of 0.01 μm. The positions of the sensors with respect to the magnetic surface are arranged by rotating the handle, and the distance between the magnet and the sensor surface is measured by the laser displacement meter (KEYENCE, LJ-G015).

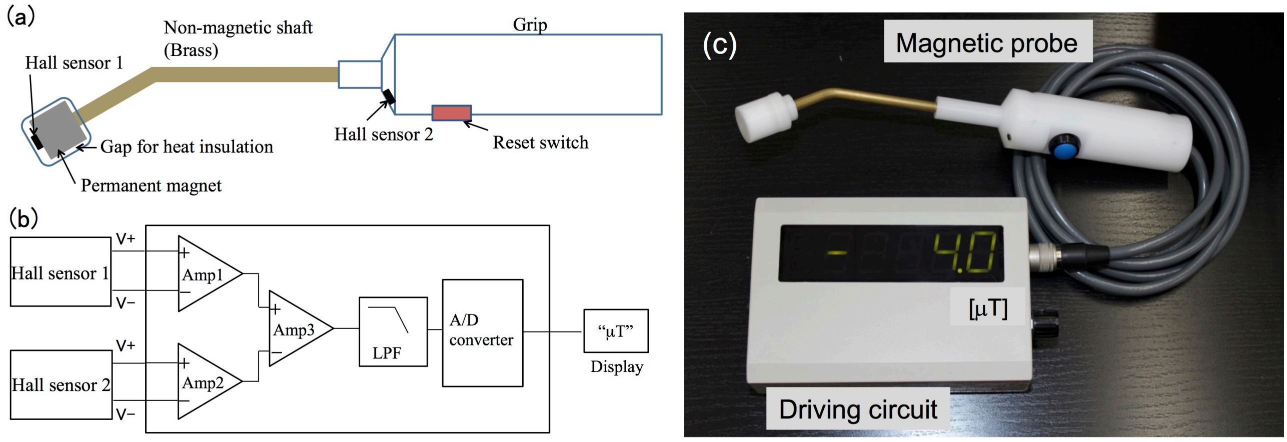

Supplementary Figure S3. Schematics of handheld magnetic probe system.

(a) The probe contains two Hall sensors. One is enclosed in the probe head to measure the magnetic signal from the SPIONs, and the other is enclosed in the probe housing. The role of the additional sensor (Hall sensor 2) is to eliminate the earth magnetic field in the Hall sensor 1. The gap between the sensor and head is approximately 1.0 mm, to isolate the ambient temperatures such as room temperature and patient's temperature. Note that the temperature variations cause thermal expansions of the magnet and probe shaft, leading to a displacement between the magnet and the sensor. However, this displacement is not large enough to change the sensitivity of the probe. The reset button located in the grip is used to cancel the variations in the measured baseline due to the thermal drifts. (b) electric circuit for detecting magnetic signals and cancelling of earth magnetic fields. The signals detected by the Hall sensors 1 and 2 are amplified by Amp. 1 and 2, respectively, and the amplified signal is amplified by a differential amplifier (Amp. 3). The maximum gain from the amplifiers is approximately 80 dB. The signal is input to the A/D convertor via a low-pass filter, and is displayed quantitatively in terms of μT. In addition, the probe generates sounds whose frequencies and beep periods are proportional to the strengths of the measured magnetic fields, to notify the detection of the SLNs. (c) Newly developed handheld magnetic probe for the clinical tests in breast cancer patients. The length and weight of the probe are approximately 300 mm and 308 g, respectively. The size of the driving circuit is 140 × 200 × 80 mm and its weight is 1.1 kg.
